# Supplementary material for: Grant reviewer perceptions of the quality, effectiveness, and influence of panel discussion
Source: Res Integr Peer Rev. 2020 May 15;5:7. doi: 10.1186/s41073-020-00093-0 (PMC7229595; doi:10.1186/s41073-020-00093-0)
Supplement: Supplementary file 2 — Additional file 2:. Supplemental Table 1 - Comparison of Answers of Early Versus Late Respondent Groups. [file 41073_2020_93_MOESM2_ESM.docx]

| **Question** | **Early Responses (N=398)** | **Late Responses (N=273)** |
| --- | --- | --- |
| Q1. Did the grant application discussions facilitate reviewer participation? | **92% [90%-95%]** | **93% [89%-96%]** |
| Q2. How useful were the grant application discussions in clarifying differing reviewer opinions? | **2.0 [1.9-2.1]** | **2.0 [1.9-2.1]** |
| Q3. Was the format and duration of the grant application discussions sufficient to allow the non-assigned reviewers to cast well informed merit scores? | **80% [76%-84%]** | **77% [72%-82%]** |
| Q4. How useful was the Chair in facilitating the application discussions? | **2.0 [1.9-2.1]** | **2.0 [1.9-2.1]** |
| Q5. Did the grant application discussions affect the outcome? | **2.0 [1.9-2.1]** | **2.0 [1.9-2.1]** |
| Q6. Did the grant application discussions promote the best science? | **2.0 [1.9-2.1]** | **2.0 [1.9-2.1]** |

Median and percentage values from responses to the above questions are listed for both early respondents (pre-reminder) and late respondents. 95% confidence intervals are displayed in brackets.
